# A panel study of occupational exposure to fine particulate matter and changes in DNA methylation over a single workday and years worked in boilermaker welders

**DOI:** 10.1186/1476-069X-12-47

**Published:** 2013-06-11

**Authors:** Molly L Kile, Shona Fang, Andrea A Baccarelli, Letizia Tarantini, Jennifer Cavallari, David C Christiani

**Affiliations:** 1 Oregon State University, College of Public Health and Human Sciences, 15 Milam, Corvallis, OR 97331, USA; 2 Harvard School of Public Health, 665 Huntington Avenue, Boston, MA 02115, USA; 3 Center of Molecular and Genetic Epidemiology, Department of Environmental and Occupational Health, University of Milan, Milan, Italy; 4 University of Connecticut, School of Medicine, Community Medicine & Health Care, 263 Farmington Avenue, Farmington, CT 06030, USA

**Keywords:** DNA methylation, PM2.5, iNOS, Welders, LINE-1, Alu, Boilermakers

## Abstract

**Background:**

Exposure to pollutants including metals and particulate air pollution can alter DNA methylation. Yet little is known about intra-individual changes in DNA methylation over time in relationship to environmental exposures. Therefore, we evaluated the effects of acute- and chronic metal-rich PM_2.5_ exposures on DNA methylation.

**Methods:**

Thirty-eight male boilermaker welders participated in a panel study for a total of 54 person days. Whole blood was collected prior to any welding activities (pre-shift) and immediately after the exposure period (post-shift). The percentage of methylated cytosines (%mC) in LINE-1, Alu, and inducible nitric oxide synthase gene (*iNOS*) were quantified using pyrosequencing. Personal PM_2.5_ (particulate matter with an aerodynamic diameter ≤ 2.5 μm) was measured over the work-shift. A questionnaire assessed job history and years worked as a boilermaker. Linear mixed models with repeated measures evaluated associations between DNA methylation, PM_2.5_ concentration (acute exposure), and years worked as a boilermaker (chronic exposure).

**Results:**

PM_2.5_ exposure was associated with increased methylation in the promoter region of the *iNOS* gene (β = 0.25, SE: 0.11, p-value = 0.04). Additionally, the number of years worked as a boilermaker was associated with increased *iNOS* methylation (β = 0.03, SE: 0.01, p-value = 0.03). No associations were observed for Alu or LINE-1.

**Conclusions:**

Acute and chronic exposure to PM_2.5_ generated from welding activities was associated with a modest change in DNA methylation of the *iNOS* gene. Future studies are needed to confirm this association and determine if the observed small increase in *iNOS* methylation are associated with changes in NO production or any adverse health effect.

## Background

Boilermakers are skilled welders who cut and weld metal plates. They use a variety of welding technologies including oxyacetylene gas torches, gas tungsten arc welding, shielded metal arc welding, or gas metal arc welding. The extreme heat produced by welding generates a complex mixture of gases, aerosols and particulate matter. This molten mixture condenses into ultrafine and fine particulate matter with an aerodynamic diameter ≤ 2.5 μm (PM_2.5_) that is easily inhaled [1]*.* Welding fumes are rich in metal oxides and can contain antimony, beryllium, cadmium, chromium, cobalt, copper, iron, lead, manganese, mercury, molybdenum, nickel, vanadium, and zinc [2,3] although the exact composition of welding fumes will depend on many factors including base metal characteristics and type of welding.

Epidemiological studies show that chronic exposure to welding fumes are associated with respiratory health effects including cardiovascular disease, asthma, bronchitis, lung function changes, and increased risk of lung cancer [4-10]. While the mechanisms linking welding fume exposure to adverse health outcomes is not fully understood, previous studies implicate several biological pathways for welding fume toxicity including oxidative stress [11], systemic inflammation [12-14], and alterations in cardiac autonomic responses [4,15,16]. It is also possible that welding fumes alter epigenetic mechanisms and subsequently gene expression. Data from previous studies shows that many of the metals detected in welding fumes including nickel, lead, cadmium, chromium, beryllium, and arsenic are associated with altered DNA methylation [17-27]. There is also evidence that exposure to particulate matter air pollution is associated with altered DNA methylation [28-31]. Changes in DNA methylation have been observed in subjects with cardiovascular disease and cancer [32]. Thus, it is possible that epigenetic changes may provide a potential mechanism by which welding fumes are linked to adverse biological effects.

We, therefore, hypothesized that exposure to particulate matter generated from welding activities can alter DNA methylation. This hypothesis was tested in boilermakers who were previously recruited for a panel study designed to evaluate the cardiopulmonary health effects of welding fumes [4,15]. We used existing data to explore the relationship between exposure to PM_2.5_ and DNA methylation over time because individuals provided blood samples collected both pre- and post-work shift, had personal PM_2.5_ occupational exposure measurements, and detailed work histories. Specifically, we hypothesized that exposure to PM_2.5_ over a single work shift would be associated with changes to DNA methylation, as well as, from cumulative exposure based on years worked as a welder.

We employed a candidate gene approach and measured DNA methylation in short interspersed nucleotide elements (Alu) and long interspersed nucleotide elements (LINE-1). These two repetitive elements make up approximately 55% of the human genome and are heavily methylated to suppress their expression [33]. Repetitive elements are activated during conditions of cellular stress and de-methylation of LINE-1 and Alu elements increase their activity as retrotransposable sequences [34-36]. Additionally, we quantified DNA methylation in the inducible nitric oxide synthase gene (*iNOS*, also known as *NOS2*, Genbank accession number AF017634) which is involved in the production of nitric oxide and plays an important role in a variety of cardio-pulmonary processes including asthma [12], chronic obstructive pulmonary disease [37], and cardiovascular health [38].

## Methods

### Participant selection

The Institutional Review Board at the Harvard School of Public Health approved the study protocol and informed consent was obtained prior to participation. The details of participant selection and study recruitment have been described previously [4,15]. This analysis used samples collected from 38 individuals who had archived whole blood samples available for DNA extraction. Briefly, boilermaker construction workers were monitored on a high-exposure welding day and low-exposure non-welding days at the Union welding school in 2003 and 2008 as part of a panel study on welding fumes and cardiovascular risk. On non-welding days, participants performed office work in a large enclosed break room adjacent to the welding room. Per the initial study design, boilermakers were allowed to participate on multiple occasions in each observation period. We observed that two boilermakers participated in both 2003 and 2008 and three boilermakers participated twice on different days in 2008. Not all participants opted to provide a post-shift blood sample. Therefore, this analysis utilized 40 blood samples collected in the morning prior to the start of a work-shift (pre-shift) and 38 blood samples were collected in the afternoon after all work activities had been completed (post-shift). Also, twenty-four individuals provided two blood samples on the same observation day (pre- and post-shift). The most common type of welding was manual metal arc welding on mild (manganese alloys) and stainless steel (chromium and nickel alloys) bases. All welding was performed in a room outfitted with 10 workstations, each with local exhaust ventilation.

### Data collection

A modified American Thoracic Society questionnaire was used to collect information on socio-demographic information, smoking history, occupational history and medical history [39].

### Exposure assessment

All individuals wore personal gravimetric particulate samplers over the duration of the work shift using a KTL cyclone (GK2.05SH, BGI Incorporated, Waltham, MA) with an aerodynamic diameter ≤2.5 μm used in line with a pump drawing 3.5 L/min of air. The inlet tubing was secured to the participant’s shoulder in the breathing zone area. Exposure to PM_2.5_ was measured over a six-hour welding shift or equivalent non-welding period as previously described by Cavallari et al. [4]. All samples were blank corrected. Two individuals were missing PM_2.5_ data. Air quality monitoring was performed on the same day as blood sample collection.

### DNA methylation

Whole blood was collected in the morning before any welding activity (pre-shift) and in the afternoon at the end of the work shift (post-shift) and frozen at -80°C. Blood samples were collected at the same times each day to account for the potential influence of circadian rhythm. DNA was extracted using Puregene DNA solutions (Qiagen, Valencia, CA, USA). DNA methylation was quantitatively measured on bisulfite-treated DNA using PCR-Pyrosequencing following the methods described by Tarantini et al. [28]. The primers and sequence that was analyzed for each marker are presented in Table 1. Samples were analyzed in duplicate and the average methylation, expressed for each DNA locus as the percentage of methylated cytosines (%mC) over the sum of methylated and unmethylated cytosine, was used in the statistical analysis. The coefficient of variation for each DNA locus was 0.009, 0.006, and 0.008 for LINE-1, *iNOS,* and Alu, respectively. All samples were subjected to a quality control check incorporated in the software which evaluates the bisulfite conversion rate of any cytosine not followed by a guanine. Five pre-shift and four post-shift blood samples failed pyrosequencing quality control for *iNOS* and were subsequently excluded from the analysis.

**Table 1:** Primers and location of CpG sites that were quantified by pyrosequencing

**Sequence ID**	**Forward Primer**	**Reverse Primer**	**Sequencing Primer**	**Sequence analyzed**
	**(5′ to 3′)**	**(5′ to 3′)**	**(5′ to 3′)**	
Global methylation analysis				
Alu	Biotin-TTTTTATTAAAAATATAAAAATT	CCCAAACTAAAATACAATAA	AATAACTAAAATTACAAAC	**G/A**C/T**G/A**C/T**G/ A**CCACCA
LINE-1	TTTTGAGTTAGGTGTGGGATATA	Biotin-AAAATCAAAAAATTCCCTTTC	AGTTAGGTGTGGGATATAGT	TT**C/T**GTGGTG**C/T**GT**C/T**G
Gene-specific methylation analysis				
*iNOS*	TTGGATGGTATGGGGTGAGTAT	Biotin-TACCCAATCCCCTCATCAA	GTGTGTTTATAATTTTGTAG	**C/T**GAGT**C/T**GAAAATTGAGGTTT**C/T**GG

Nucleotides where DNA methylation was measured are in bold text.

### Statistical analysis

The average %mC was calculated for 3 CpG sites in LINE-1, 3 CpG sites in Alu, and 4 CpG sites in *iNOS*. Linear mixed effects regression models with a repeated statement to account for repeated measurements on the same subjects were used to evaluate the relationship between %mC and PM_2.5_; as well as, the relationship between %mC and the number of years each individual worked as a boilermaker. Separate models were constructed for LINE-1, Alu, and *iNOS*. In the models that looked at cross-shift PM_2.5_ exposure, pre-shift blood %mC for each gene was included as a predictor in the models to control for individual factors that may influence methylation. Whereas, pre-shift blood %mC was not included in the models that evaluated the relationship between the number of years a person reported working as a boilermaker and methylation. All models were adjusted for smoking (yes/no), wearing a respirator (yes/no), and age. Since age was highly collinear with the number of years a person worked as a boilermaker (σ_spearman_=0.57), we used the residuals from a regression model that had age as the dependent variable and years worked as a boilermaker to adjust for age (age_RS_) in models that looked at the association between methylation and chronic exposure. All analyses were performed with SAS version 9.2.

## Results

This panel study consisted of 38 adult males who provided a total of 54 measurement events (Table 2). The average age of the participants at first enrollment was 35.6 years (range: 21.3 to 61.0 years) and they had worked an average of 6.8 years as a boilermaker (range: 1–35 years). The majority of participants reported their race as white (85.3%) and 31.6% reported that they currently smoked. In 13 instances during sampling, a respirator was used by the worker. The mean work shift exposure for PM_2.5_ over the 54 measurement events was 1.06 mg/m^3^ (median: 0.52 mg/m^3^, range: 0.02-3.41 mg/m^3^, average duration of exposure, 303.2 minutes; SD = 84.1 minutes; range: 61–339 minutes). Eighty-two percent of the blood samples were collected from individuals who welded during the day of collection and 18% were collected from individuals who were present at the union hall but did not participate in any welding activities. The average percentage of methylated cytosines at baseline for Alu, LINE-1 and *iNOS* was 25.5%, 85.3%, and 97.5%, respectively (Table 3).

**Table 2:** Description of selected characteristics in 38 boilermakers at the time of their first recruitment

	***N***	**Mean (SD) or Percent**	**Range**
*Cohort characteristics*			
Age (years)^a^	*34*	36.0 (12.0)	21.3-61.0
Males	*38*	100%	
Race			
Caucasian	*31*	83.3%	
African-American	*5*	11.1%	
Hispanic	*2*	5.6%	
Current smoker			
Yes	*12*	30.6%	
No	*26*	69.4%	
Years worked as boilermaker^b^	*36*	6.8 (9.4)	1 - 35
*Exposure characteristics*^*c*^			
		Median (IQR) or Percent	
PM_2.5_ exposure (mg/m^3^)^c^	*54*	0.52 (1.34)	0.02-3.41
Weld day samples^d^			
Yes	*44*	81.5%	
No	*10*	18.5%	
Used respirator			
Yes	*13*	24.1%	
No	*41*	75.9%	

^a^Age at initial participation in the study. Missing age = 2.

^b^Missing self-reported number of years working as a boilermaker = 2.

^c^Descriptive statistics based on 54 observation days.

^d^Missing personal PM_2.5_ measurements = 2.

**Table 3:** Description of DNA methylation for the repeated blood sample measurements

	**Baseline**					**Post-shift**				
	***N***	**Mean**	**SD**	**Min**	**Max**	***N***	**Mean**	**SD**	**Min**	**Max**
Alu (%mC)	*40*	25.5	0.3	25.0	26.0	*38*	25.5	0.4	23.8	25.9
LINE-1 (%mC)	*40*	85.3	0.9	82.0	87.3	*38*	85.4	0.6	84.3	86.9
*iNOS* (%mC)	*36*	97.5	0.8	95.2	99.2	*34*	97.3	0.6	96.2	98.7

Linear mixed effect models evaluated the association between PM_2.5_ exposure across the work shift and methylation in blood samples collected post-shift (Table 4). We observed that PM_2.5_ exposure was associated with increased methylation within the promoter region of the *iNOS* gene (β = 0.25, SE: 0.11, p-value = 0.04) in models that adjusted for *iNOS* methylation measured pre-shift, current smoking status, age, and respirator usage. When the analysis was restricted to only those 25 individuals who actually performed welding activities on the observation day, the association between PM_2.5_ and *iNOS* methylation was marginally greater (β = 0.35, SE: 0.11, p-value = 0.006). This suggested that individuals who were directly exposed to welding fume PM_2.5_ had a stronger response compared to individuals who were exposed to background PM_2.5_ within the practice hall. When the cohort was further restricted to exclude the five individuals who wore respirators on the observation day, the association between PM_2.5_ and *iNOS* methylation was fractionally greater (β = 0.38, SE: 0.14, p-value = 0.02) which further supported the association between metal-rich welding fume PM_2.5_ and modest increased methylation in the promoter region of the *iNOS* gene. Overall, these results suggested that for every 1 mg/m^3^ increase in PM_2.5_, *iNOS* methylation increased an average of 0.25% (95% CI: 0.01 – 0.48%). No association was observed between PM_2.5_ and Alu (β = 0.05, SE: 0.07, p-value = 0.47) or LINE-1 (β = −0.12, SE: 0.10, p-value = 0.28).

**Table 4:** **Association between PM**_**2.5 **_**exposure across the work shift (mg/m**^**3**^**) and methylation of *Alu*, *LINE-1 *and *iNOS *measured in whole blood collected post-shift**

		**Unadjusted regression**^**1**^				**Adjusted regression**^**2**^		
	***N***	**β**	**SE**	**p-value**	***N***	**β**	**SE**	**p-value**
Global DNA Methylation								
Alu (%5mC)	*37*	0.02	0.07	0.73	*35*	0.05	0.07	0.47
LINE-1 (%5mC)	*37*	-0.05	0.10	0.61	*35*	-0.12	0.10	0.28
Gene specific DNA Methylation								
*iNOS* (%5mC)	*29*	0.22	0.10	0.04	*27*	0.25	0.11	0.04

^1^ Adjusted for DNA methylation in the sample pre-shift.

^2^ Adjusted for DNA methylation in the sample pre-shift, currently smoking (yes), age, and wearing a respirator.

To evaluate the association between DNA methylation and chronic exposure to PM_2.5_, linear mixed effect models evaluated the relationship between the number of years each participant worked as a boilermaker and DNA methylation (Table 5). These models suggested that chronic exposure to welding fumes, as characterized by years worked as a boilermaker, were associated with small increases in methylation of the promoter region within the *iNOS* gene (β = 0.03, SE: 0.01, p-value = 0.03) after adjusting for current smoking status and age_RS_. No association between years worked as a boilermaker and methylation in Alu (β = -0.004, SE: 0.004, p-value = 0.28) or LINE-1 (β = 0.02, SE: 0.02, p-value = 0.28) was observed. We further restricted this analysis to only pre-shift samples to avoid the potential for acute work shift exposures to influence the observed associations and observed similar effects (Table 6).

**Table 5:** **Association between years as a boilermaker and methylation of Alu, LINE-1 and *iNOS *measured in all whole blood samples**

		**Unadjusted regression**				**Adjusted regression**^**1**^		
Global DNA Methylation	*N*	β	SE	p-value	*N*	β	SE	p-value
Alu (%5mC)	*77*	-0.002	0.004	0.66	*73*	-0.01	0.005	0.05
LINE-1 (%5mC)	*77*	0.005	0.01	0.64	*73*	0.01	0.01	0.28
Gene specific DNA Methylation								
*iNOS* (%5mC)	*70*	0.02	0.01	0.006	*66*	0.03	0.01	0.02

^1^ Adjusted for currently smoking (yes), white blood cell count, and age.

**Table 6:** **Association between years as a boilermaker and methylation of Alu, LINE-1 and *iNOS *measured in only in pre-shift whole blood samples**

		**Unadjusted regression**				**Adjusted regression**^**1**^		
Global DNA Methylation	*N*	β	SE	p-value	*N*	β	SE	p-value
Alu (%5mC)	*42*	-0.001	0.004	0.13	*40*	-0.02	0.005	0.002
LINE-1 (%5mC)	*42*	0.02	0.02	0.24	*40*	0.03	0.02	0.15
Gene specific DNA Methylation								
*iNOS* (%5mC)	*38*	0.03	0.01	0.007	*36*	0.04	0.02	0.02

^1^ Adjusted for currently smoking (yes) and age.

Considering that smoking was common in this population and might be an effect modifier of *iNOS* methylation, an interaction term between years worked as a boilermaker and current smoking was also evaluated in the model. This interaction term was not significant which indicated that there was no effect modification by smoking status (β_interaction_ = 0.10, SE: 0.09, p-value = 0.31).

## Discussion

We observed that occupational exposure to fine particulate matter generated from welding activities measured across a single work-shift was associated with modest increases in DNA methylation in the promoter region of the inducible nitric oxide synthase gene in whole blood DNA. The number of years worked as a boilermaker was also associated with modest increased DNA methylation in the *iNOS* gene. However, we did not observe any relationship between acute exposure to welding fume PM_2.5_ or years worked and DNA methylation in two repeated elements, Alu and LINE-1. These findings suggest that methylation of the *iNOS* gene may be mutable over short periods of time.

DNA methylation regulates gene expression with increased methylation leading to gene silencing. Biologically, it is plausible that *iNOS* could be influenced by exposure to PM_2.5_ generated from welding activities. Animal models show that acute exposure to welding fumes produce reactive oxygen species and inflammatory cytokines including tumor necrosis factor-alpha and interleukin-1 beta [5,40]. In turn, these immunologic and inflammatory factors stimulate inducible nitric oxide synthase which produces nitric oxide (NO) which can constrict vascular beds, produce hypertensive responses, regulate nonspecific host defenses, and modulate inflammation [41,42]. These effects appear to depend on the PM composition. For instance, PM derived from urban sources diminished NO production, whereas, respirable cristobalite, fine particulate matter with enriched polycyclic aromatic hydrocarbons, aerosols derived from wildfire smoke, and traffic-generated PM increased NO production [43-45]. There is also evidence that welding fume PM_2.5_ influences NO production. Prior epidemiological research by our group showed that the fractional concentration of expired breath nitric oxide decreased after five days of exposure to welding-related PM_2.5_ and that the decreases in expired NO were associated with the soluble metal fraction in the PM_2.5_[46]_._ This inhibition in exhaled NO would be consistent with diminished expression of genes involved in the synthesis of NO.

Other environmental epigenetic studies have also investigated the association between particulate matter air pollution and DNA methylation in the *iNOS* gene. For instance, Tarantini *et al.* reported that particulate matter with aerodynamic diameters < 10 μm (PM_10_) was associated with a 0.61% decrease in methylation in the *iNOS* gene measured in whole blood DNA across a 3-work day period in a cohort of 63 foundry workers [28]. Another study reported that 7-day average ambient PM_2.5_ was associated with decreased *iNOS* methylation (0.30% decrease in *iNOS* per 5 μg/m^3^ increase in ambient PM_2.5_) in DNA extracted from buccal cells collected from 940 children who resided in the Los Angeles area [47]. Both of these studies observed an inverse association between increased particulate matter exposure and decreased DNA methylation in the *iNOS* gene. Whereas, our study observed a positive association between increased PM_2.5_ and increased DNA methylation in the *iNOS* gene. It is not clear why the direction of the effect differs between our study and the two previous studies but it is worth noting we measured different CpG sites within the promoter region of the *iNOS* gene and perhaps different CpG sites even within the same promoter region respond differently to environmental stimuli. Also, the composition of the particulate matter likely differed between the studies. It is also possible there was an uncontrolled confounding by smoking and/or second hand smoke exposure. As such, it is difficult to draw direct comparisons between the three studies.

We recognize that there are several limitations to our study. Methylation is tissue specific and we used DNA extracted from whole blood which is a mixture of different white blood cell types. Ultrafine particles diffuse across the alveoli and enter the bloodstream where they exert a direct effect on white blood cells by triggering an immune response. Since we did not measure white blood cell counts or measure DNA methylation in specific white blood cell sub-populations we cannot rule out that the increased *iNOS* DNA methylation was produced by systemic inflammation from inhaling fine particulate matter [13,14,48]. Another limitation of the study was that we did not collect samples that would allow for measurement of gene expression or production of nitric oxide to determine if the methylation changes in iNOS had any biological impact.

However, our study had several strengths including personal measurements of PM_2.5_ across the work shift which minimizes the potential for misclassification of exposure. We used pyrosequencing which is a very sensitive method and provides a quantitative measurement of DNA methylation. It is also worth noting that the blood samples were stored for several years in a cold storage facility at −80°C prior to analysis which likely reduced DNA yield but not DNA quality.

## Conclusions

We observed a positive relationship between occupational exposure to fine particulate matter generated from welding activities and a modest increase in DNA methylation in the promoter region of the iNOS across a single work shift. We also observed a positive association between the number of years a person reported working as a boilermaker and increased DNA methylation in the iNOS gene. These results suggest that DNA methylation may be altered by PM_2.5_ over a short period of time. Further studies are needed that collect multiple measurements over longer periods of time to more fully understand the dynamic relationship between welding fume exposures and DNA methylation. These studies should also measure blood cell mixture to determine if the changes in DNA methylation are true epigenetic effects or a function of inflammation.

## Abbreviations

Alu: A family of short interspersed elements; CpG: Cytosine-guanine nucleotide sequence; iNOS: Inducible nitric oxide synthase; LINE-1: Long interspersed nucleotide elements; LOD: Limit of detection; mC: Methylated cytosines; NO: Nitric oxide; PM2.5: Fine particulate matter with an aerodynamic diameter <2.5 μm; PM10: Fine particulate matter with an aerodynamic diameter <10 μm; PM: Particulate matter; SD: Standard deviation.

## Competing interests

The authors declare that they have no competing interests.

## Authors’ contributions

MLK – conceived the study, conducted the data analysis, and drafted the manuscript. SF – conducted the sample collection and exposure assessment, participated in the design of the study and helped with the manuscript preparation. AB – participated in the DNA methylation analsysis and helped with the manuscript preparation. LT – performed the DNA methylation analysis.JC – conducted the sample collection and exposure assessment, participated in the design of the study, and helped with the manuscript preparation.DCC –conceived of the study, and participated in its design and coordiation, and helped with the manuscript preparation. All authors read and approved the final manuscript.
